# High Fidelity Copy Number Analysis of Formalin-Fixed and Paraffin-Embedded Tissues Using Affymetrix Cytoscan HD Chip

**DOI:** 10.1371/journal.pone.0092820

**Published:** 2014-04-03

**Authors:** Yan P. Yu, Amantha Michalopoulos, Ying Ding, George Tseng, Jian-Hua Luo

**Affiliations:** 1 Department of Pathology, University of Pittsburgh School of Medicine, Pittsburgh, Pennsylvania, United States of America; 2 Department of Statistics, University of Pittsburgh School of Medicine, Pittsburgh, Pennsylvania, United States of America; Istituto dei tumori Fondazione Pascale, Italy

## Abstract

Detection of human genome copy number variation (CNV) is one of the most important analyses in diagnosing human malignancies. Genome CNV detection in formalin-fixed and paraffin-embedded (FFPE) tissues remains challenging due to suboptimal DNA quality and failure to use appropriate baseline controls for such tissues. Here, we report a modified method in analyzing CNV in FFPE tissues using microarray with Affymetrix Cytoscan HD chips. Gel purification was applied to select DNA with good quality and data of fresh frozen and FFPE tissues from healthy individuals were included as baseline controls in our data analysis. Our analysis showed a 91% overlap between CNV detection by microarray with FFPE tissues and chromosomal abnormality detection by karyotyping with fresh tissues on 8 cases of lymphoma samples. The CNV overlap between matched frozen and FFPE tissues reached 93.8%. When the analyses were restricted to regions containing genes, 87.1% concordance between FFPE and fresh frozen tissues was found. The analysis was further validated by Fluorescence In Situ Hybridization on these samples using probes specific for BRAF and CITED2. The results suggested that the modified method using Affymetrix Cytoscan HD chip gave rise to a significant improvement over most of the previous methods in terms of accuracy in detecting CNV in FFPE tissues. This FFPE microarray methodology may hold promise for broad application of CNV analysis on clinical samples.

## Introduction

Genome abnormalities are the hallmark of human malignancies [1]. These include chromosome deletion, amplification, translocation, inversion and isochromosome formation. Analysis of genome abnormality is critical in making diagnosis of human malignancies, congenital birth defects and a variety of inheritable diseases. Array comparative genome hybridization (aCGH) or Affymetrix SNP array has been frequently applied to clinical samples to examine loss of heterozygosity and to detect amplification or deletion of genome fragments in the chromosomes [2]–[8]. The current methodologies using aCGH or Affymetrix SNP6.0 require high quality genome DNA from fresh frozen tissues. However, most of the samples for pathological evaluation are formalin-fixed and paraffin-embedded (FFPE) tissue blocks. Suboptimal and high background results are obtained when tissues from FFPE tissue blocks are analyzed due to the fragmenting nature of genome DNA in FFPE tissues. The low quality of genome copy number analysis from FFPE tissues practically precludes the application of whole genome copy number variation (CNV) analyses in clinical setting. Thus, a new method that can reproducibly generate high quality CNV analysis from FFPE tissues is needed to make high throughput genome CNV analysis applicable to clinical setting.

Lymphoma is one of the human malignancies that are frequently associated with large number of structural genome abnormalities. The classification and treatment of lymphomas are based on their genotypes in the tumor cells. Thus, lymphoma is an ideal human malignancy to investigate whether FFPE tissue is suitable for CNV analysis using Affymetrix Cytoscan HD chip. In this report, we describe a method that is adapted to the genomic DNA extracted from FFPE tissues to prepare the DNA cocktail for Affymetrix Cytoscan HD analysis. To validate this method, frozen genome DNA samples from matched lymphoma cases were also analyzed on Affymetrix Cytoscan HD chips. The results showed close overlaps in CNV profiles between FFPE and matched frozen tissues.

## Materials and Methods

### Tissue samples

Fresh frozen tissues of eight cases of human malignant lymphomas, including 5 diffused large B cell lymphomas, 2 follicular lymphomas and 1 T cell non-Hodgkins lymphoma, were obtained from clinical services. These tissues were dissected to have at least 70% purity of tumor cells. The study was approved by University of Pittsburgh Medical Center Quality Insurance Committee and Institutional Review Board, and exempted from informed-consent. The matched FFPE tissues from the same patients were also frozen sectioned onto slides, fixed and dehydrated with 100% ethanol and similarly micro-dissected to obtain tumor cells. Karyotyping analyses were performed on all these cases to detect chromosome abnormalities.

### Affymetrix CytoScan HD chip analysis of copy number variation of tumor cells

For macrodissected frozen tissues, DNA was extracted using QIAamp blood and tissue kit (Qiagen, Valencia, CA). Five hundred nanograms of genome DNA were digested with Nsp1 for 2 hours at 37°C. The digested DNA was purified and ligated with primer/adaptors at 16°C for 12–16 hours. Amplicons were generated by performing PCR using primers provided by the manufacturer (Affymetrix, CA) on the ligation products using the following program: 94°C for 3 min, then 35 cycles of 94°C 30 second, 60°C for 45 sec and 65°C for 1 minute. This was followed by extension at 68°C for 7 min. The PCR products were then purified and digested with DNAseI for 35 min at 37°C to fragment the amplified DNA. The fragmented DNA was then labeled with biotinylated nucleotides through terminal deoxynucleotide transferase for 4 hours at 37°C. Two hundred fifty micrograms of fragmented DNA were hybridized with a pre-equilibrated Affymetrix chip Cytoscan HD chip at 50°C for 18 hours. The procedures of washing and scanning of CytoscanHD chips followed the manuals provided by Affymetrix, Inc. Cel files were generated from AGCC software from Affymetrix, Inc. (Santa Clara, CA). For FFPE tissues, micro-dissected tumor cells were treated with xylene for 12 hours. DNA was then extracted using QiaAmp FFPE DNA extraction kit. Gel purification of DNA sizes ranging from 200 to 1000 bp was performed. Two hundred fifty nanograms of purified DNA was then digested with NSP1, and similarly processed as frozen tissues.

### Statistical analysis

Sixteen cel files were analyzed with Genotyping console for quality control analysis. Samples with QC call above 80% were admitted into the analysis. To analyze CNV, cel files were imported into Chromosome Analysis Suite 1.2 (Affymetrix, Inc) to generate copy number from raw intensity. For frozen tissues, Cytoscan HD files from fresh frozen tissues of 380 healthy individuals provided by Affymetrix were used as a baseline. For FFPE tissues, Cytoscan HD files from FFPE tissue of 100 healthy individuals from Affymetrix were used. Deletions or amplifications of genomes were analyzed by first limiting to the regions with p-value less than 0.05/total number of regions detected, i.e. family-wise error rate (FWER) is controlled using Bonferroni's correction [9]. The selected regions were filtered by limiting to the regions with at least 25 markers and 500 kb. For genome fragment gain determination, a mean of >2.3 for autosomal chromosomes or >1.5 for sex chromosomes of male was required, while for genome fragment loss determination, a mean of <1.7 for autosomal chromosomes or <0.5 of sex chromosomes of male was required. Loss of heterozygosity was not analyzed due to lack of matched normal tissues.

### Fluorescence In-situ Hybridization (FISH)

Tissue slides (5 microns) were placed in 2×SSC at 37°C for 30 min. Slides were then removed and dehydrated in 70% and 85% ethanol for 2 min each at room temperature, and air dried. The probes for CITED2 and BRAF FISH analysis were obtained from BACPAC Resource Center, Oakland, CA. The DNA from the selected clone was extracted using Nucleobond Ax kit (Macherey-Nagel, Easton, PA). The probe was prepared by combining 7 µl of biotin-labeled genomic sequence containing CITED2 or BRAF (150 Kb)/50% formamide with 1 µl of direct-labeled CEP7 spectrum green for BRAF or CEP6 for CITED2 (Vysis, Downers Grove, IL). The probe was denatured for 5 min at 75°C. Sections of formalin-fixed tissues were denatured in 70% formamide for 3 min, and dehydrated in 70%, 85%, and 100% ethanol for 2 min each at room temperature. The denatured probe was placed on the slide, cover-slipped, sealed with rubber cement, placed in a humidified chamber and hybridized overnight at 37°C. Coverslips were removed and the slides were washed in 2×SSC/0.3% NP-40 at 72°C for 2 min. Slides were then held in phosphate buffered saline (PBS) at room temperature in the dark for 2 min. The biotin label was visualized by conjugation with Avidin-spectrum orange (Zymed, San Francisco, CA), cover-slipping and incubating in a moist chamber in the dark at 37°C for 20 min. Slides were washed 3 times for 2 min each in fresh PBS. Slides were then air-dried in the dark and counterstained with DAPI. Analysis was performed using a Nikon Optiphot-2 and Quips Genetic Workstation equipped with Chroma Technology 83000 filter set with single band exitors for Texas Red/Rhodamine, FITC and DAPI (uv 360 nm). Only individual and well delineated cells with two hybridization signals were scored. Overlapping cells were excluded from the analysis. Fifty to 100 cells per sample were scored to obtain an average of signals.

### Karyotyping analysis

Cytogenetic analysis was performed on cell cultures of lymphoma samples. The cell cultures were stimulated with phytohemagglutinin (PHA) for 72 hours, and harvested and treated briefly with a hypotonic solution. The cells were then fixed with Carnoy fixative. GTG-banding was carried out using standard protocols [10].

## Results

Affymetrix Cytoscan HD contains 2.6 million markers covering all RefSeq genes and 750K SNPs of human genome, and has >99% genotype accuracy. It has been widely applied to ascertain genome abnormalities of a variety of human diseases. However, it is rarely applied to FFPE tissues. To evaluate the usage of Cytoscan HD on clinical FFPE samples, eight cases of lymphoma FFPE tissues were micro-dissected and analyzed through Affymetrix Cytoscan HD chips. The frozen counterparts of these cases were similarly analyzed. Karyotype analyses were performed on all these cases for validation purpose. As shown in Table 1, karyotype abnormalities in 24 of 51 loci had complete matches with frozen tissue copy number analyses. Twenty-two loci from Karyotype analyses had significant overlapped regions detected by frozen tissue CNV analysis. Overall, this represents 90.2% (46/51) concordance between Karyotype and Cytoscan HD analyses. Five loci of Karyotype analyses, however, did not concord with CNV analysis from both frozen and FFPE tissues analyses. These differences may result from heterogeneity of the tumor samples that some genome abnormalities appear in only a fraction of tumor cells.

**Table 1:** Genotyping concordance between FFPE and Frozen tissues.

Case	Frozen genotype					FFPE genotype				Karyotype
	Chr	Locus	Copy	Def	Size	Locus	Copy	Def	Size	
Case 1	2	2p16.1	1	Loss	0.7 MB	2p16.1	1	Loss	0.5 MB	−2
		2q33.2-34	2.3	Gain	7.2 MB	2q33.2-34	2.3	Gain	7.2 MB	
		2q35-37.3	3	Gain	24 MB	2q35	2.4	Gain	24 MB	
	5	5q13.2-14.3	1.3	Loss	18.4 MB	5q13.2-14.3	1.4	Loss	17 MB	−5
		5q21.1-22.1	1.4	Loss	12 MB	5q21.1-22.1	1.5	Loss	10.5 MB	
		5q33.3-35.1	1.3	Loss	12.4 MB	5q33.3-35.1	1.45	Loss	11.8 MB	
	7	7p14.3-14.1	1.3	Loss	6.5 MB	7p14.3-14.1	1.5	Loss	6.7 MB	der(7)t(7;?)(p11.2;?)
		7q11.22-22.1	4	Gain	27.8 MB	7q11.22-22.1	4	Gain	24.4 MB	add(7)(q22) [8]
		7q22.1-36.3	3	Gain	38.3 MB	7q22.1-36.3	3	Gain	33.5 MB	der(7;16)(p10;q10)
	8	8p23.3-11.21	1.3	Loss	42.6 MB	8p23.3-11.22	1.4	Loss	39.6 MB	−8 [17]
		10q23.1-25.1	1.3	Loss	21.7 MB	10q23.1-25.1	1.5	Loss	21.3 MB	
	11	11p15.3-15.1	1.5	Loss	10.1 MB	11p15.4-14.3	1.5	Loss	14.2 MB	
		11p14.2-11.2	2.5	Gain	22 MB	11p14.2-11.12	2.4	Gain	23.6 MB	
	13	13q33.3-34	1.3	Loss	6.6 MB	13q33.3-34	1.3	Loss	6.7 MB	add(13)(q34) [15]
	16	16p11.2-11.1	4	Gain	0.8 MB	16p11.2-11.1	3	Gain	1.2 MB	
		16p11.1-q21	1.3	Loss	30.4 MB	16p11.1-q22.1	1.5	Loss	33 MB	−16
		16q23.1	4	Gain	2.7 MB	16q23.1	4	Gain	2.6 MB	
		16q23.1-23.2	1.2	Loss	3.7 MB	16q23.1-23.2	1.2	Loss	3.7 MB	
		16q23.2-23.3	4	Gain	1.2 MB	16q23.2-23.3	4	Gain	1.2 MB	
		16q23.3-24.1	1.1	Loss	2.5 MB	16q23.3-24.1	1.1	Loss	2.5 MB	
		16q24.1	4	Gain	2.0 MB	16q24.1	4	Gain	2.0 MB	
		16q24.1-24.3	1.2	Loss	2.8 MB	16q24.1-24.3	1.2	Loss	2.8 MB	
	17	17p13.3-13.1	2.5	Gain	8.5 MB	17p13.3-13.1	2.5	Gain	8.7 MB	−17
		17p12-q11.2	3	Gain	14.5 MB	17p12-q11.2	2.7	Gain	16.7 MB	
		17q21.33-25.33	2.7	Gain	31.8 MB	17q21.33-25.32	2.7	Gain	32.2 MB	
	X	Xp22.33-q28	2	NP	154.3 MB	Xp22.33-q28	3	Gain	154.3 MB	+X [6]
Case 2	1	1p36.33-36.21	1.4	Loss	16.7 MB	1p36.33-36.13	1.5	Loss	18.2 MB	
		1q21.2-q44	2.7	Gain	102 MB	1q21.2-q44	2.6	Gain	102 MB	der(1)(qter>1q21::p36.3>qter)
	2	2p25.3-11.2	2.8	Gain	89.1 MB	2p25.3-11.2	2.6	Gain	88.9 MB	
	6	6q21-27	1.2	Loss	58.9 MB	6q21-27	1.4	Loss	58.7 MB	
	17	17p13.3-11.2	1.3	Loss	16.9 MB	17p13.3-11.2	1.5	Loss	19.2 MB	del(17)(p11.2)
		17p11.2-q25.3	2.8	Gain	59.7 MB	17p11.2-q25.3	2.7	Gain	60.3 MB	i(17)(q10)
	18	18p11.32-q21.33	2.9	Gain	60.5 MB	18p11.32-q21.33	2.8	Gain	60.1 MB	+der(18)(t(14;18)(q32;q21) [4]
	22	22q11.22	1	Loss	0.9 MB	22q11.22	1	Loss	1.0 MB	
	X	Xp22.33-q28	3	Gain	156 MB	Xp22.33-q28	3	Gain	156 MB	+X
Case 3	1	1q24.3-42.11	2.9	Gain	55.1 MB	1q24.3-42.11	2.7	Gain	52 MB	+1, der (1)
	5	5p15.33-q35.3	2.6	Gain	180.6 MB	5p15.33-q35.3	2.6	Gain	180.6 MB	5
	8	8p23.3-21.3	1.4	Loss	22.8 MB	8p23.3-21.3	1.4	Loss	22.3 MB	del(8)(p21p23)
	9	9p24.1-23	2.5	Gain	4.7 MB	9p24.2-23	2.4	Gain	7.1 MB	add(9)(p22)
	10	10p15.3	1.4	Loss	0.8 MB	10p15.3	1.5	Loss	0.8 MB	dic(1;10)(10qter>10p15::1p13>1q25::1q21>1q32::1q25>1qter)
		10p15.1-11.21	2.5	Gain	31.6 MB	10p15.1-11.21	2.5	Gain	31 MB	
	14	14q32.33	1	Loss	0.7 MB	q32.33	1	Loss	1.0 MB	add(14)(q32)
	18	18p11.32-q11.2	3	Gain	22 MB	18p11.32-q11.2	3	Gain	22 MB	18
		18q11.2-23	4	Gain	55 MB	18q11.2-23	4	Gain	55 MB	
	19	19q13.2-13.43	2.7	Gain	18.1 MB	19q13.2-13.43	2.6	Gain	19.4 MB	
	X	Xp22.33-q28	3	Gain	156 MB	Xp22.33-q28	3	Gain	156 MB	+X
Case 4	1	1p31.1	1.2	Loss	13.7 MB	1p31.1	1.4	Loss	13.2 MB	78–79,inc[cp5]*
		1q23.3-32.1	2.3	Gain	31.4 MB	1q23.3-32.1	2.3	Gain	29.5 MB	
		1q42.2-44	1.2	Loss	17.2 MB	1q42.2-43	1.5	Loss	10 MB	
	2	2p25.3-24.3	2.7	Gain	13.3 MB	2p25.3-24.3	2.3	Gain	14.3 MB	
		2p24.3-16.3	1.5	Loss	34 MB	2p24.3-16.3	1.6	Loss	34.1 MB	
	3	3p26.3-q13.13	1.6	Loss	108.1 MB	3p25.1-12.2	1.7	Loss	66.2 MB	
	4	4p16.3-q24	1.6	Loss	106.6 MB	4p16.3-q24	1.6	Loss	104.6 MB	
		4q25-34.1	2.7	Gain	64.7 MB	4q25-34.1	2.5	Gain	65.5 MB	
		4q34.3-35.2	2.6	Gain	7.4 MB	4q34.3-35.2	2.5	Gain	9.3 MB	
	5	5p15.33-15.31	2.6	Gain	8.7 MB	5p15.33	3	Gain	0.7 MB	
		5q15-22	1.5	Loss	54.6 MB	5q23.3-33.1	1.6	Loss	22.5 MB	
	7	7p22.3-21.1	1.1	Loss	17.8 MB	7p22.3-21.1	1.4	Loss	17.3 MB	
		7q11.22-36.3	3	Gain	93.5 MB	7q11.22-36.3	2.8	Gain	93.6 MB	
	8	8p23.3-12	1.6	Loss	32.9 MB	8p23.3-12	1.6	Loss	30.9 MB	
	9	9p23.1-22.3	0.8	Loss	8.5 MB	9p23.1-22.3	1.2	Loss	8.1 MB	
		9p21.3-21.1	0.7	Loss	7 MB	9p21.3-21.1	1	Loss	6.9 MB	
		9p21.1	0.8	Loss	1 MB	9p21.1	1.3	Loss	1 MB	
		9p13.3	1	Loss	1.3 MB	9p13.3	1	Loss	1.3 MB	
		9p13.3-q21.33	0.9	Loss	54.9 MB	9p13.3-q21.33	1.3	Loss	53.2 MB	
		9q22.2-22.31	0.7	Loss	2.5 MB	9q22.2-22.31	1	Loss	2.4 MB	
		9q22.31-22.33	1.2	Loss	4.4 MB	9q22.31-22.33	ND	NA	NA	
		9q31.1-31.2	0.8	Loss	6.3 MB	9q31.1-31.2	1.1	Loss	5.6 MB	
		9q32-34.11	1	Loss	15.1 MB	9q32-34.11	1.3	Loss	17 MB	
	10	10p15.3-12.1	3	Gain	28.1 MB	10p15.3-12.1	2.8	Gain	28.4 MB	
		10p11.22	0.3	Loss	2.6 MB	10p11.22	0.7	Loss	3.6 MB	
	11	11q13.1-13,4	1.6	Loss	8.7 MB	11q13.1-13,4	1.7	Loss	7 MB	
	13	13q12.11-34	1.2	Loss	96.7 MB	13q12.11-34	1.3	Loss	96.7 MB	
	14	14p11.2	0.3	Loss	0.5 MB	14p11.2	0.7	Loss	0.6 MB	
		14q32.32	0.7	Loss	0.6 MB	14q32.32	1.2	Loss	0.6 MB	
	17	17p13.3-13.2	1.2	Loss	5.9 MB	17p13.3-13.2	1.5	Loss	5.8 MB	
		17p13.2-13.1	3	Gain	1.7 MB	17p13.2-13.1	3	Gain	0.9 MB	
		17p13.1-11.2	1.2	Loss	14.4 MB	17p13.1-11.2	1.3	Loss	13.4 MB	
		17q11.1-25.3	3.2	Gain	55.7 MB	17q11.1-25.3	2.9	Gain	54.2 MB	
	20	20q11.22-13.33	3.2	Gain	30.4 MB	20q11.22-13.33	2.7	Gain	31.1 MB	
	21	21q11.2-22.3	1.5	Loss	33.1 MB	21q21.2-22.3	1.7	Loss	23 MB	
Case 5	10	10q22.3-24.32	1.4	Loss	21.7 MB	10q22.3-24.32	1.5	Loss	22.3 MB	del(10)(q24q26)
	13	13q14.13-22.1	1.5	Loss	28.4 MB	13q14.13-22.1	1.5	Loss	27.4 MB	de(13)(q14q22)
Case 6	1	1p11.2-q21.2	2.6	Gain	27.4 MB	1p11.2-q21.2	2.5	Gain	33.3 MB	
	4	4p16.3-q36.2	1.4	Loss	190.9 MB	4p16.3-q36.2	1.6	Loss	188.2 MB	−4
	5	5p15.33-q11.2	3.2	Gain	58.8 MB	5p15.33-q11.2	2.5	Gain	57.5 MB	+5, i(5)(p10)
	6	6p25.3-21.1	3.1	Gain	45 MB	6p25.3-21.1	2.5	Gain	45.3 MB	
		6p12.3-q27	1.4	Loss	121 MB	6p12.3-q27	1.6	Loss	97 MB	−6
	7	7p22.3-14.1	2.7	Gain	41.9 MB	7p22.3-14.1	2.5	Gain	45.2 MB	
	13	13q31.1-34	3	Gain	35.8 MB	13q31.1-34	2.5	Gain	35.8 MB	add(13)(q34)
	18	18q21.11-21.33	3.2	Gain	28.4 MB	18q21.11-21.33	2.7	Gain	28.2 MB	add18(q21)
		18q21.33-23	1	Loss	17.5 MB	18q21.33-23	1.3	Loss	17.6 MB	
	20	20p13-q11.23	2.6	Gain	38.1 MB	20p13-q11.23	2.5	Gain	38.1 MB	
Case 7	1	1p12-q44	2.4	Gain	128.8 MB	1p12-q44	2.4	Gain	128.2 MB	+1,add(1)(p32)
	2	2p25.3-16.1	1.6	Loss	58.6 MB	2p25.3-16.1	1.6	Loss	58.1 MB	−2
		2p16.1-14	3.5	Gain	8.7 MB	2p16.1-14	3	Gain	8.7 MB	
	3	3p14.1-13	3.9	Gain	7.6 MB	3p14.1-13	3.5	Gain	7.6 MB	3
		3q25.2-26.1	1.7	Loss	8.3 MB	3q25.2-26.1	1.7	Loss	6.0 MB	
	4	4p16.3-q35.2	1.6	Loss	190.9 MB	4p16.3-q35.2	1.6	Loss	190.9 MB	−4
	6	6p26.3-11.2	2.4	Gain	58.3 MB	6p26.3-11.2	2.5	Gain	57.3 MB	+6,i(6)(p10)
	7	7p22.3-36.3	2.4	Gain	159 MB	7p22.3-36.3	2.4	Gain	159 MB	7
	10	10p16.3-15.2	1.6	Loss	3.6 MB	10p16.3	1.7	Loss	1.8 MB	dic(1;10)(q10;p13)
	17	17p13.3-q11.1	1.6	Loss	25.6 MB	17p13.3-q11.1	1.6	Loss	25.9 MB i(17)(q10)	i(17)(q10)
	20	20p12.3-12.1	1.5	Loss	5.9 MB	20p12.3-12.1	1.6	Loss	5.9 MB	−20
		20q11.1-13.33	1.6	Loss	33.3 MB	20q11.1-13.33	1.6	Loss	33.1 MB	
Case 8	1	1p36.33-12	1.6	Loss	96.7 MB	1p36.33-12	1.7	Loss	96.7 MB	i(1)(q10)
		1q12-44	2.5	Gain	102.3 MB	1q12-44	2.4	Gain	102.1 MB	
	2	2p25.3-25.2	1.7	Loss	6.3 MB	2p25.3-25.2	1.7	Loss	5.0 MB	
		2p21-13.3	2.4	Gain	22.1 MB	2p16.3-13.3	2.4	Gain	17.2 MB	add(2)(p11.2)
		2q23.1-37.3	2.4	Gain	44.3 MB	2q23.1-37.3	2.3	Gain	37.1 MB	
	3	3p26.3-12.1	1.7	Loss	86.1 MB	3p26.3-12.1	1.7	Loss	84.1 MB	del(3)(p13p25)
	4	4p16.3-q35.2	1.6	Loss	188.5 MB	4p16.3-q35.2	1.7	Loss	188.5 MB	−4
	6	6q25.2-27	2.8	Gain	17.3 MB	6q25.2-27	2.5	Gain	18.5 MB	
	7	7q21.11-36.3	2.5	Gain	78.3 MB	7q21.11-36.3	2.4	Gain	79.2 MB	add(7)(q36)
	8	8p23.3-q23.1	1.6	Loss	108.8 MB	8p23.3-q21.11	1.7	Loss	78 MB	del(8)(q13q24)
	9	9p24.3-q34.3	2.5	Gain	140.8 MB	9p24.3-q34.3	2.4	Gain	140.8 MB	9
	10	10p15.3-q26.3	1.7	Loss	136.3 MB	10p15.3-q26.3	1.7	Loss	136.3 MB	−10
	11	11q12.1-13.5	1.7	Loss	19.2 MB	11q12.1-13.5	ND	NA	NA	−11
	15	15q11.2-26.3	1.7	Loss	79.6 MB	15q11.2-26.3	1.7	Loss	74 MB	−15
	18	18p11.32-q23	1.6	Loss	77.8 MB	18p11.31-q23	1.7	Loss	72.3 MB	−18
	X	Xp22.33-21.2	1.5	Loss	30.1 MB	Xp22.33-21.2	ND	NA	NA	XXX
		Xp21.2-q21.33	2.5	Gain	63 MB	Xp21.2-q28	2.5	Gain	133 MB	

NP-normal ploidy; ND-Not detected; NA-Not applicable; Def-copy number variation definition;

*-No analysis was reported due to low number of cells survived.

**Figure 1:**
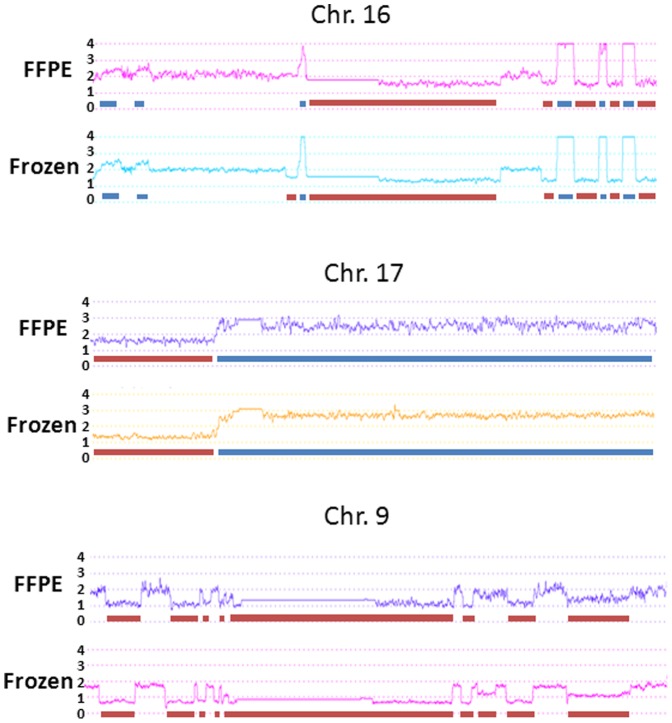
Histograms of matched FFPE and Fresh frozen samples on selected chromosomes. Top panel: FFPE and frozen histograms of chromosome 16 of Case 1; Mid panel: FFPE and frozen histograms of chromosome 17 of Case 2; Lower panel: FFPE and frozen histograms of chromosome 9 of Case 4. Red bar denotes deletion. Blue bar denotes amplification.

Most of the clinical samples are in the form of formalin-fixed and paraffin-embedded tissue blocks. The dependence of array analysis on fresh frozen tissues significantly limits its application in clinical setting. To investigate whether FFPE tissues are suitable for cytoscan HD analysis, DNA from the matched FFPE tissue samples was extracted. Similar Affymetrix Cytoscan HD analyses were performed. Cel files of FFPE tissues from 100 normal individuals were used as baseline to calculate the copy number of genome fragments of these FFPE samples. CNV was determined by p<0.05 with at least 25 markers and a minimal length of 500 Kb. As shown in Tables 1–2 and figure 1, the concordant rates based on CNV length between arrays from the matched FFPE and frozen tissues ranged from 82% to over 99%. Seven of these FFPE blocks are over 1 year old and one is 3 years. The average concordant rate for 1 year old samples is 94.1%, while the rate for the 3 year old sample is 92%. This suggests that the quality of the assays is stable, and is probably not adversely affected by the age of the fixed tissues for at least 1–3 years. When CNV calling was limited to regions that cover at least one gene, we found that 482 genome segments were either deleted or amplified in at least one of the 16 samples. Among these segments, 68 of 86 segments that were determined as deletion in FFPE samples matched the same callings from fresh frozen tissue counterparts, while 352 of 365 segments that were determined as amplification matched those from the fresh tissues (Table 3). The results confirmed a strong correlation between FFPE and fresh frozen tissues (87.1%, Pearson correlation coefficient = 0.72, p<2.2×10^−16^). Interestingly, when CNV results of FFPE were matched with Karyotype studies, the results were extremely similar to those between frozen CNV and Karyotype. There is no statistically significant difference in terms of accuracy. In fact, a slight improvement (25/51 complete match versus 24/51 frozen) was seen due to detection of X chromosome gain in a case that was missed in frozen tissue.

**Table 2:** CNV Overlaps between Frozen tissues and matched formalin-fixed paraffin-embedded tissues.

Case	Loss by Frozen	loss by FFPE	Overlap	Gain by Frozen	Gain by FFPE	Overlap
Case 1	170.4 MB	170.3 MB	91.70%	180.8 MB	177.3 MB*	92.70%
Case 2	93.4 MB	97.1 MB	95.60%	311.3 MB	311.3 MB	99.60%
Case 3	24.3 MB	24.1 MB	98.80%	523.1 MB	523.1 MB	98.60%
Case 4	614.9 MB	545 MB	82.10%	334.9 MB	327.5 MB	94.90%
Case 5	50.1 MB	49.7 MB	96.80%	N/D	N/D	N/A
Case 6	329.4 MB	302.8 MB	91.90%	275.4 MB	283.4 MB	95.90%
Case 7	326.2 MB	321.7 MB	98.40%	362.7 MB	360.8 MB	99.60%
Case 8	829.4 MB	734.9 MB	88.70%	468.1 MB	527.9 MB	82%

N/D-Not detected; N/A-Not applicable.

*Excluding X chromosome.

**Table 3:** Correlation of CNV callings between FFPE and matched fresh frozen tissues by segment number.

CNV	fresh gain	fresh loss
FFPE loss	68	18
FFPE gain	13	352

P<2.2×10^−16^; Pearson correlation coefficient = 0.72.

**Figure 2:**
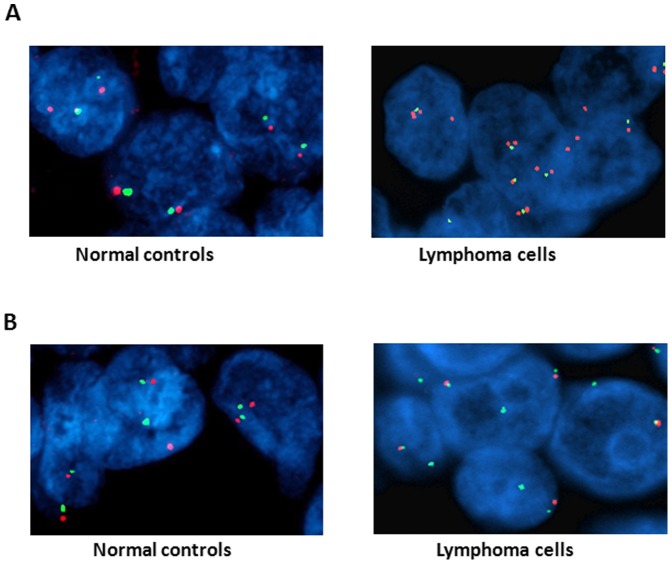
FISH validation of copy number variation detected by Cytoscan HD analysis. (A) Representative images of FISH analysis using probes specific for BRAF (7q34, red) and centromere of chromosome 7 (green). Left, normal diploid control, right-case 4 lymphoma (BRAF amplified). (B) Representative images of FISH analysis using probes specific for CITED2 (6q23.3, red) and centromere of chromosome 6 (green). Left, normal diploid control, right-case 2 lymphoma (CITED2 deleted).

To validate the CNV analysis at individual gene level, FISH assays were performed on all 8 cases of lymphoma using probes specific for BRAF and CITED2. Four samples of lymphoma were found to have gain of BRAF gene by CNV analyses in both frozen and FFPE tissues. Each of these 4 cases was found to have similar amplification of BRAF in the FISH assays, while the other 4 CNV neutral samples were found to have copy number near diploid condition in FISH. The concordant rate between the CNV calls from FFPE or frozen samples and the FISH results from the matched samples reaches 100% (8/8, Table 4 and figure 2). CITED2, a transcription modulator essential for glycolytic metabolism for adult hematopoietic stem cells and potential tumor suppressor [11], [12], was found deleted in 2 cases of lymphoma by CNV analysis in frozen tissues. On the other hand, CNV analysis in the matched FFPE tissues suggests loss of CITED2 in only one sample. FISH assays using a probe specific for CITED2 found loss of one copy of CITED2 gene in both cases where deletions were also indicated by Cytoscan HD chip analyses of frozen tissues (Table 4 and figure 2). However, 55% (117/212) of the counted cells in one of the cases (case 6) in the FISH assay do not contain deletion of CITED2. The large dilution by the diploid cells in this sample may result in a negative result in FFPE CNV analysis.

**Table 4:** Correlation of CNV callings and FISH on BRAF and CITED2.

Genes	Cases	CN by frozen tissue	CN by FFPE	CN by FISH
BRAF	1	3	3	2.8*
	2	Unchanged	Unchanged	2.1
	3	Unchanged	Unchanged	1.9
	4	3	3	2.9*
	5	Unchanged	Unchanged	2.2
	6	Unchanged	Unchanged	1.9
	7	3	2.5	2.7*
	8	3	3	3.1*
CITED2	1	Unchanged	Unchanged	2.1
	2	1	1	1.3*
	3	Unchanged	Unchanged	1.9
	4	Unchanged	Unchanged	2
	5	Unchanged	Unchanged	2.2
	6	1.4	Unchanged	1.6*
	7	Unchanged	Unchanged	1.8
	8	Unchanged	Unchanged	2

CN-copy number;

*-p<0.01.

Despite the high level statistical CNV concordance between FFPE and frozen tissues, a moderately higher level of fluctuation was readily detected in FFPE array analysis (figure 1). The average of copy number for genome deletion for FFPE tissues is 1.44. This magnitude of deletion is significantly less than that from frozen tissues (1.29, p = 6.7×10^−11^). As a result, CNV analysis from FFPE tissues could be less sensitive. Despite this mild drawback, excellent CNV concordance between FFPE and frozen samples was evident in most of the CNV regions, particularly those CNV loci with large DNA fragment abnormalities (figure 1).

## Discussion

The current methodologies to detect chromosome abnormalities include Karyotyping using Giemsa staining of chromosomes, high throughput array analysis of single nucleotide polymorphism and DNA copy number, and whole genome sequencing. The first two are the most commonly used methods to determine the copy number of genome fragments, while the last one might be highly precise but expensive. Array genome copy number analysis offers a high resolution alternative to Karyotyping assay. However, its clinical application is limited due to the requirement of high quality DNA. Most clinical specimens, however, are stored in the form of formalin-fixed and paraffin-embedded tissue blocks. The DNA from FFPE is highly fragmented and cross-linked. This produces a significant challenge in using FFPE tissues for high resolution genotyping analysis. Our method using FFPE tissues to analyze chromosomes shows an average of concordance of 93.8% between FFPE and fresh frozen tissues. It suggests that FFPE Cytoscan HD analysis is readily applicable to clinical setting.

Studies using FFPE tissues to analyze copy number variation had been peviously attempted on Affymetrix 10K and SNP6.0 chips, GenomePlex aCGH, Illumina beadArray and Agilent 244K chips (Table 5) [13]–[19]. However, many of these studies lack direct validation using other methodologies. In one study, high noise level on CNV analysis was found in FFPE samples when using Affymetrix 6.0 chip. Only 53% concordant rate was found between the matched frozen and FFPE samples [15]. In another study, only selected concordance analyses were performed on selected regions of FISH and Agilent 244K chips using FFPE tissues [14]. The study concluded complementary roles of between aCGH and FISH analyses. Both analyses were performed on FFPE tissues. Recently, a FFPE OncoScan service was developed by Affymetrix Inc to use Molecular Inversion Probe technology to detect CNV of oncogenic hot spots [20], [21]. The signal-to-noise ratios were reduced even using FFPE tissues more than 5 years old. However, these studies lacked direct validation comparison between FFPE and matched frozen tissues. Thus, the fidelity of CNV callings was not determined. Another study using optimization of universal linkage system labeling to analyze 3 cases FFPE and matched frozen tissues. They found a good correlation in 2 cases (Pearson correlations 0.54–0.58), but found poor correlation in the other case [22]. To our knowledge, this is the first report that shows high concordance in CNV analysis between FFPE and frozen tissues using Affymetrix Cytoscan HD chip. The CNV results from FFPE not only matched well with those from frozen tissues, but were also largely validated by cytogenetic karyotyping and FISH analyses. The Cytoscan HD FFPE analysis holds promise for being widely used in solid tumor and hematological diseases diagnosis. It may be also useful in differential diagnoses of hereditary diseases.

**Table 5:** Comparison of methods analyzing CNV using FFPE tissue.

	Thompson et al	Little et al	Tuefferd et al	Lips et al	Oosting et al	Soroush et al	Yu et al
Platform	Affymetrix	GenomePlex	Affymetrix	Illumina	Illumina/Affymetrix	Agilent/Affymetrix	Affymetrix
Array Type	10K	aCGH	SNP 6.0	BeadArray	BeadArray/10K	aCGH/SNP6.0	CytoscanHD
Number of Loci	10000	5623	1.85 million	5861	5861/10000	40161/1.85 million	2.6 million
CNV FFPE vs. Frozen	NE	NE	53%	NA	NE	NA	93.80%
CNV FFPE vs. karyotype	NE	NE	NE	NA	NE	NA	91%
FISH validation rate	NE	NE	NE	NA	NE	NA	93.80%

NE-Not examined; NA-Not applicable.
